# Increased prevalence of eating disorders as a biopsychosocial implication of food allergy

**DOI:** 10.1371/journal.pone.0198607

**Published:** 2018-06-26

**Authors:** Barbara Wróblewska, Anna Maria Szyc, Lidia Hanna Markiewicz, Magdalena Zakrzewska, Ewa Romaszko

**Affiliations:** 1 Institute of Animal Reproduction and Food Research of Polish Academy of Sciences, Department of Immunology and Food Microbiology, Olsztyn, Poland; 2 Non-Public Health Care Clinic of Allergy “ALLERGICA”, Olsztyn, Poland; 3 Non-Public Health Care Clinic “ATARAX”, Olsztyn, Poland; Dana-Farber Cancer Institute, UNITED STATES

## Abstract

**Introduction:**

The study evaluates the impact of biopsychosocial factors involved in food allergy (FA) on the prevalence of eating disorders (ED). For the 5-year follow-up studies, 75 participants (aged 1–14 years) with early-onset FA and 81 healthy peers were included.

**Method:**

Participants were diagnosed with FA using antibody/cytokine content immunoassay tests. Medical history, including BMI *z*-scores, was completed using data obtained in response to a validated allergic questionnaire that incorporated the SCOFF and EAT-8 screening questionnaires for ED. FA was confirmed if total IgE was elevated, specific sIgE to food allergens exceeded 0.7 kUA/L and if manifestations were observed. Screening for ED was considered positive if two or more SCOFF and EAT-8 items were confirmed.

**Results:**

In the FA^+^ group, 50% of female participants and 6.7% of their healthy female peers reported ED. An ED^+^ result was more frequent in FA^+^ individuals than in their healthy peers (*p* = 0.046) although the association is weak. In the FA^+^/ED^+^ group, 25.3% of the participants were underweight, and 14.7% were overweight compared to their peers where this reached respectively 4.2% and 2.8% (*p*<0.005). 74% of the FA^+^/ED^+^ individuals reported elimination diet implementation and only 15% declared it was medically consulted. The prevalence of ED in the FA^+^ male group was consistently correlated with lack of confidence in FA issues (r = 0.5424) and in the FA^+^ female group with applied medical procedures (r = 0.7069; *p*<0.005).

**Conclusion:**

These findings suggest that participants with FA especially struggling with lack of confidence in FA issues and those following an uncontrolled, restrictive elimination diet are more prone to food aversion and ED than their healthy peers. Applied procedures are necessary, and their neglect is associated with FA deterioration; however, the possibility of ED and biopsychosocial implications development should not be underestimated.

## Introduction

According to the World Health Organization (WHO), a constant growth of morbidity in allergic diseases among the populations of children has been observed. Currently, allergies are the third most frequently diagnosed chronic disease in the European population [1]. Food allergy (FA) due to the similarity of its symptoms with the food intolerance, a non-allergic detrimental reaction, is often mistaken by society and patients. Symptoms observed in both type of diseases, especially in infancy or early childhood, include feeding disorders (FD) clinically manifested by recurrent vomiting, spitting, burping, colic and diarrhoea. This may result in an aversion to food intake, weight faltering and nutrient deficiencies [2]. Moreover, FD can also result in consolidation of improper dietary habits and further eating disorders (ED) such as *anorexia nervosa*, *bulimia nervosa*, *orthorexia nervosa* or overeating, leading to overweight and obesity [3]. The aetiology of both FA and ED is multifactorial. Several biosociodemographic factors and a patient’s individual predispositions contribute to their development [4].

The most popular protocol applied for the treatment of food allergy and intolerance is elimination diet (ELD). It aim at reducing the risk of antigen intake. Such an approach deteriorate the quality of life of patients and their families. Moreover, it was stated that the proportion of children in developed populations, such as Japanese or Polish, who unnecessarily and unsuccessfully eliminate food, may reach almost 50% [5, 6]. Strict adherence to an ELD and potential undesirable consequences of diet derogation may cause severe adverse reactions to food and psychological burden. Some reports suggest that this protocol may lead to development of compulsive behaviours [7–9]. An additional problem is the incorrectly composed ELD without counselling by a dietician or doctor. It may result in a nutritional deficiency, malnutrition and metabolic disorders. It is particularly dangerous during the period of intensive development of children. The deficiency of such components as calcium, vitamin D, vitamin B complex and DHA can aggravate the psychological condition of patients and, in extreme cases, can trigger development of depression [10].

Anti-allergy steroid and antihistamine drugs may also have a significant impact on FD and ED development in allergic diseases. These drugs are commonly used to minimize allergic manifestation [11]. There are reports of body weight side effects of anti-allergic drugs (AD) that block histamine H1-H4 receptors to relieve allergy symptoms [12,13]. It should be emphasized that histamine–an inflammatory and allergic mediator–may also be consumed through diet [14, 15] and is physiologically released in the brain, where it is responsible for appetite regulation and taste perception, among other functions. The cumulation of histamine exacerbate of allergic and intolerance symptoms and hence may increase the intake of AD which are over-the-counter drugs and their use is out of medical control. Comorbidity of related central nervous system side effects is also observed in steroid therapy, potentially resulting in a hunger/satiety state imbalance, swelling and weight gain [16]. Puberty period of children especially coincides with increased endocrine disruption, which promotes an exacerbation of allergic diseases. Application of one or both therapies is indisputable in some cases; however, their biopsychosocial implications, including ED, are being observed in younger and younger patients [17]. Currently, dietary restraint has been reported by 35% of 5-year-old girls [18] and its coincidence with FA, diagnosed in 1–17% of preschool children, is probable. What is important in the course of both this diseases, the most common biophysiological abnormality in childhood is an imbalance of regulatory activity of the immune system [19, 20].

Several studies have analysed the prevalence of ED among patients with diet-related diseases, it has not been widely discussed in the context of FA yet, and most of the research is based on surveys and self-reported manifestation. The aim of this study was to compare the prevalence of symptoms of ED in underage patients with immunologically confirmed, long-duration FA and in healthy peers, as well as to assess which of the additional sociodemographic, environmental and biological variables can have an influence on this phenomenon.

## Method

### Participants

The underage participants of the study originated from the group of patients and the control group recruited for the national project entitled “Influence of fermented cow’s milk products displaying reduced antigenicity on the immunological response of Warmia and Mazury region’s patients with food allergy with consideration of genetic aspects” [No N312 311939], undertaken in the north-eastern region of Poland. Cross-sectional analyses accounted for the repeated measures obtained from involved individuals (N = 180) during onset at the age of 0–14 years and follow-up after 5 years (N = 156; response rate: 87%). All participants (mean inclusion age 5.9, SD 3.7 years; 55.6% female) were enrolled by collaborating allergologists between the years 2010 and 2014. During the recruitment phase of the study, an allergic group of people (N = 90) and an equitable group of healthy participants were recruited. FA^+^ group was characterized by positive results of immunological serum parameters (total IgE > age norm and specific IgE >0.7 kUA/L) and presence of typical allergic manifestation. The control group, recruited from preschool children, was characterized by negative results of immunological serum parameters (total IgE < age norm and specific IgE <0.7 kUA/L) and no allergic manifestation. Detailed inclusion-exclusion criteria and assessments for the participants in this study were specified in **S1 Table** in Supporting Information. Also the more comprehensive characteristics of the studied underage population is provided in **S2 Table** in Supporting Information. All procedures have been approved by the local ethical committee (Case No. 2/2010) and followed in accordance with the standards of the Helsinki Declaration. Written informed consent was obtained from all parents or statutory guardians of participants.

### Immunological analyses

The total immunoglobulin E levels of the patients were measured using ImmunoCAP (Phadia AB, Uppsala, Sweden) during the recruitment period (IgE-*r*), whereas the ECLIA method using Elecsys (Roche Diagnostics, Poland) was used during the 5-year follow-up phase (IgE-*f*). Blood parameters (absolute number of lymphocytes in the blood and albumin content) were determined using a Cobas analyser equipped with Cobas MIRA Plus (Roche Diagnostics). Follow-up measurements and blood parameter determination were carried out by the following authorized laboratories: Prof. Dr. Stanisław Popowski Regional Specialized Children’s Hospital in Olsztyn and The Nicolaus Copernicus Municipal Polyclinical Hospital in Olsztyn. Food specific serum immunoglobulins E (sIgE) were determined using the EUROLINE Paediatric profile for allergy diagnosis (EUROIMMUN AG, Lübeck, Germany). Food allergy was confirmed if concentration of IgE specific to food allergens exceeded 0.7 kUA/L. Positive serum tests were confirmed by percutaneous skin tests (Allergopharma-Nexter, Germany) in cooperating medical units. Total immunoglobulin G level and serum IL-2, IL-4 and INF-γ cytokine content were determined using the enzyme linked immunosorbent assay (ELISA) method with commercially available kits (BD Bioscience, USA). Sensitivity of the assays was 7.8–500 pg/mL for IL-2 and IL-4, 4.7–300 pg/mL for INF-γ and 0.021–15 ng/mL for IgG.

### Assessments

Data collected for all patients included age, gender, weight and height, and BMI *z*-scores standardized and calculated for age and gender [21, 22, 23]. Based on BMI *z*-score body weight status classification was described in **S1 Table** in Supporting Information. Medical history complemented with demographics data were submitted by parents/ caregivers and/or by the patients themselves, both in cooperation with the allergologist. The standardized questionnaire for the allergic study corresponded to the validated EuroPrevall study questionnaire was supplemented with additional questions about dietary habits, body image attitude, parental and children allergy problems, and FA issue confidence. To assess the frequency of eating disorders (ED^+^), the SCOFF [24], and later, the reference EAT-8 [25] questionnaires, were used. In the analysed population, EAT-8 scores were significantly correlated with SCOFF scores (r = 0.83; p<0.001). The description of both used tests is specified in **S1 Text** in Supporting Information. Questions were focused on the core symptoms of *anorexia nervosa* and *bulimia nervosa* as well as body image and dieting behaviour [26]. SCOFF questions remained a highly effective screening instrument for detection of ED comorbidity with different diseases [27].

### Statistics

Absolute frequencies and percentages for qualitative variables and means with standard deviation (SD) for quantitative variables were calculated. For quantitative data with outliers and skewed distribution, the central tendency was presented as the median with (1^st^-3^rd^) interquartile range (IQR). Comparisons of nominal variables among groups of subjects were made using the Fisher exact test and the χ^2^-test; post hoc comparison of percentages was made using Bonferroni’s (*P*_B_) corrected significance threshold of 0.008. Comparison of quantitative variables among more than two different groups was made using the parametric analysis of variance (ANOVA) for normally distributed data and the Tukey test as a parametric post hoc test or the non-parametric Kruskal-Wallis test in case of absence of homoscedasticity. A T-test or non-parametric Mann-Whitney U-test for the comparison of male *vs*. female was used. For the prospective analyses of variables between groups, the Friedman test and T^2^ Tamhane’s test or the Cochran test were used, respectively, for the quantitative and qualitative variables. The prevalence of ED (SCOFF^+^ and EAT-8^+^ results) was presented as percentages with 95% confidence intervals (CIs). To analyse the association of disordered eating (FA^-^/ED^+^), allergy to food (FA^+^/ED^-^) or combined factors (FA^+^/ED^+^) with additional analysed covariates, the groups were compared with children without respective symptoms (FA^-^/ED^-^). Correlation between quantitative variables was evaluated using the Pearson’s correlation coefficient (r) and that between nominal variables was evaluated using the Spearman's rank correlation coefficient. Factors significantly related to ED^+^ were used in multivariate logistic regression analysis, which was applied to assess the role of different independent explanatory variables in occurrence of ED symptoms in allergic groups. Odd ratios (ORs) with 95% CIs were calculated using a backward procedure with the K-fold cross validation method. For the statistical significance evaluation of the model, the Wald likelihood ratio test (LR test) was used. Adjustments were made to gender, W/A *z*-score category and the two groups of characteristics selected as dominant in principal component analysis. Explanatory variables in the first model (Adj.1) were new-born feeding type, parent-reported food allergy, and residence, whereas in the second model (Adj.2), the variables were used allergy diagnostic method, dominant allergy symptoms, and therapy implementation. All tests were two sided, and a *P* value less than 0.05 or an OR with 95% CI not overlapping the 1.00 value was considered statistically significant. Data analysis was performed using the statistical package STATISTICA 12 software (StatSoft, Cracow, Poland) equipped with Medical Tests Panel for multivariate analyses.

## Results

### Characteristics of participants’ groups and prevalence of ED in context of allergy

#### Age-sex structure associations

Of the 90 allergic patients enrolled in this study, 10% suffered from allergy to a single food-origin allergen (FA^+^S), 29% suffered from polyallergy to various food allergens (FA^+^P), and 61% suffered from mixed polyallergy to aero- and food allergens (FA^+^M) **(Table 1)**. The allergy profile in the FA^+^ group was significantly altered in the 5-year period. In the follow-up group of participants, 5% suffered from FA^+^S, 47% suffered from FA^+^P, and 48% suffered from FA^+^M. The age-sex structure of allergic and control population did not differ significantly in the recruitment phase or in the follow-up phase of the observation study although some of the participants had to be excluded (**S2 Table**). Nevertheless, a tendency (*p =* 0.173) towards more frequent eating disturbance in females than in males was initially observed only in allergic group, whereas in the follow-up phase, this phenomenon was statistically significant (*p =* 0.007) **(Table 2)**. A significantly higher susceptibility of FA participants for ED development in the youngest [<6 years old] (*p =* 0.0041) and in the oldest group [15–18 years old] (*p =* 0.0027) was observed compared to that of participants in the control group.

**Table 1:** Demographic and descriptive parameters of subjects during the recruitment and the 5-year follow-up phases of the study, according to health status categories.

• Groups by health status categories • Subgroupscategorized according to:	Recruitment phase of the study					5 year follow up studies					*P*-value ^c^
	FA^-^	Type of food allergy (FA^+^)			*P*-value ^a^	Control Group (FA-)	Type of food allergy (FA^+^)			*P*-value ^a^	
		FA^+^S	FA^+^P	FA^+^M			FA^+^S	FA^+^P	FA^+^M		
	n = 90 (3)	n = 9	n = 26	n = 55		n = 81	n = 6	n = 35	n = 34		
**Age** ‒ mean [SD]	5.5 [3.4]	5.2 [2.3]	6.5 [3.9]	6.4 [3.9]	0.869 ^b^	11.1 [3.9]	11.3 [2.0]	12.7 [4.2]	10.2 [3.6]	0.883^b^	0.370 ^d^
**Gender**: female ‒ n (%)	50 (55.6)	5 (55.6)	14 (53.8)	36 (65.5)	0.642	45 (55.6)	4 (66.6)	17 (48.6)	16 (47.2)	0.629	0.089
**Birth method:** natural ‒ n (%)	63 (70)	7 (77.8)	19 (73)	43 (78.2)	0.738	57 (70.4)	4 (66.6)	27 (77.1)	24 (70.6)	0.902	0.160
**New-born feeding type** ‒ n (%) Exclusive breastfeeding	36 (40.0)	2 (22.2)	7 (27.0)	16 (29.1)	**0.008**	31 (38.3)	1 (16.6)	6 (17.1)	8 (23.5)	**0.005**	**0.012 ↓**
Infant formulas	16 (17.8)	5 (55.6)	14 (53.8)	20 (36.4)		15 (18.5)	4 (66.6)	19 (54.3)	15 (44.1)		0.321
Mixed breastfeeding & formulas	38 (42.2)	2 (22.2)	5 (19.2)	19 (34.5)		35 (43.2)	1 (16.6)	10 (28.6)	11 (32.4)		**0.048 ↓**
**Residence** ‒ n (%) City	63 (70.0)	9 (100)	22 (84.6)	42 (76.4)	0.133	48 (59.3)	4 (66.6)	19 (54.3)	29 (85.3)	0.041	**<0.001 ↓**
Village	27 (30.0)	0 (0.0)	4 (16.4)	13 (23.6)		33 (40.7)	2 (33.4)	16 (45.7)	5 (14.7)		**0.013 ↑**
**Positive family history** **of atopy** ‒ n (%)	25 (27.8)	5 (55.6)	20 (76.9)	12 (18.8)	**<0.001**	20 (24.7)	4 (66.6)	17 (48.6)	12 (35.3)	0.023	**0.024 ↑**
**Dominating symptoms** ‒ n (%) Gastrointestinal manifestation	0 (0.0)	6 (66.7)	21 (80.8)	21 (38.2)	0.014	0 (0.0)	6 (100.0)	15 (42.9)	18 (52.9)	0.790	**0.012 ↓**
Respiratory manifestation	0 (0.0)	2 (22.2)	1 (3.8)	10 (18.2)		0 (0.0)	0 (0.0)	9 (25.7)	9 (26.5)		**0.013 ↑**
Skin/mucosal manifestation	0 (0.0)	1 (11.1)	4 (15.4)	24 (43.6)		0 (0.0)	0 (0.0)	11 (31.4)	7 (20.6)		**<0.001 ↓**
**Diagnostic methods** ‒ n (%) Skin Prick Tests	0 (0.0)	7 (77.8)	10 (38.5)	25 (45.5)	0.321	0 (0.0)	4 (66.6)	10 (28.6)	18 (52.9)	0.118	**0.011 ↓**
Serum tests	0 (0.0)	1 (11.1)	4 (15.4)	4 (7.3)		0 (0.0)	1 (16.6)	19 (54.3)	15 (44.1)		**0.002 ↑**
Oral challenge	0 (0.0)	1 (11.1)	2 (7.7)	2 (3.6)		0 (0.0)	1 (16.6)	6 (17.1)	1 (2.9)		0.960
**Therapy** ‒ n (%) ELD	1 (1.1)	8 (88.9)	17 (65.4)	26 (40.6)	**<0.001**	4 (4.9)	6 (100.0)	29 (82.9)	13 (38.2)	**0.007**	**0.045 ↑**
Antihistamine drugs	0 (0.0)	6 (66.7)	6 (23.1)	29 (45.3)	**<0.001**)	0 (0.0)	4 (66.6)	19 (54.3)	22 (64.7)	**0.007**	**0.045 ↓**
Steroids	0 (0.0)	1 (11.1)	3 (11.5)	18 (28.1)	**<0.001**	0 (0.0)	1 (16.6)	9 (25.7)	13 (38.2)	**<0.001**	0.159
Desensitization	0 (0.0)	0 (0.0)	0 (0.0)	9 (14.1)	**0.004**	0 (0.0)	0 (0.0)	5 (14.3)	7 (20.6)	**0.005**	0.083
**Additional dieting** ‒ n (%)	15 (16.7)	1 (9.0)	4 (15.4)	14 (25.5)	0.493	15 (18.5)	3 (50.0)	17 (48.6)	15 (44.1)	**0.001**	**0.045 ↑**
**Body weight status** ^e^‒ n (%) Underweight	13 (14.4)	3(33.3)	8 (30.8)	19 (34.6)	**<0.001**	9 (11.1)	3 (50.0)	16 (45.7)	13 (38.2)	**<0.001**	0.181
Normal	71 (78.9)	5 (55.6)	13 (50.0)	23 (41.8)		64 (79.0)	3 (50.0)	8 (22.9)	17 (50.0)		**0.016 ↑**
Overweight	6 (6.7)	1 (11.1)	5 (19.2)	13 (23.6)		8 (9.9)	0 (0.0)	11 (31.4)	4 (11.8)		0.080

FA^-^–control group, FA^+^S–allergy to single food-origin allergen, FA^+^P–polyallergy to various food-origin allergens, FA^+^M–mixed polyallergy to aero- and food-origin allergens. ELD–elimination diet. Data are presented as the means [SD] or percentages. Significant associations are marked in bold. Arrows: ↑– significant increase, ↓– significant decrease.

^a^ *p* value of the Fisher’s exact test for comparison between groups with Bonferroni’s (*P*_B_) corrected significance threshold of 0.008.

^b^ p value of the Kruskal-Wallis test for quantitative data for comparison between groups.

^c^ *p* value of the Q Cochran test for dependent groups in follow-up studies for qualitative data.

^d^ *p* value of the Friedman test for dependent groups in follow-up studies for quantitative data.

^e^ Classified according to the WHO classification [21, 22].

**Table 2:** Prevalence of disordered eating ED^+^ (stated if both SCOFF and EAT-8 were positive) in groups categorized by age, gender, new-born feeding type, weight status, type of allergy syndromes, diagnostic method and implemented therapy.

Health status: Subgroups categorized according to:	FA-	Type of food allergy (FA^+^)			
		FA^+^S	FA^+^P	FA^+^M	P-value ^a^
**Gender** Female	5.0 (0.2; 7.9)	10.5 (3.4; 16.6)	13.8 (6.2; 21.3)	17.5 (13.6; 21.4)	**0.007**
Male	7.9 (1.8; 14.0)	13.2 (8.3; 17.9)	18.4 (9.6; 27.2)	14.5 (6.5; 22.5)	
**Age:** < 6	6.3 (0.7;14.3)	12.5 (5.9; 20.9)	18.8 (5.2; 32.2)	25.0 (6.5; 43.5)	0.012
7–14	6.2 (0.9; 11.5)	3.7 (0.4; 7.8)	9.9 (3.4; 16.4)	16.0 (8.2; 24.1)	
>15	8.5 (1.3; 15.7)	0.0 (0.0; 0.0)	17.0 (7.4; 26.7)	23.6 (4.8; 42.4)	
**New-born feeding type** Exclusive breastfeeding	5.8 (0.7; 12.7)	2.2 (0.2; 5.3)	10.1 (3.8; 16.4)	8.9 (2.9; 14.9)	**0.008**
Infant formulas	12.3 (1.4; 26.0)	8.0 (0.7; 15.4)	24.2 (6,2; 42.2)	16.0 (0.5; 31.5)	
Mixed breast & formulas	6.7 (1.5; 11.9)	5.9 (0.6; 12.4)	11.8 (2.9; 20.7)	23.8 (12.0; 35.6)	
**Body weight status** ^b^ Underweight	12.1 (0.8; 23.4)	18.2 (4.8; 31.5)	15.2 (2.7; 27.6)	31.2 (7.1; 55.4)	0. 016
Normal	4.1 (0.1; 8.1)	5.2 (0.7; 9.6)	17.5 (9.9; 25.1)	13.4 (6.6; 20.2)	
Overweight	11.1 (0.5; 24.7)	7.4 (0.2; 15.9)	18.5 (2.8; 34.2)	14.8 (0.6; 23.8)	
**Blood parameters** Lymphopenia <1 thous./μL	3.7 (0.9; 7,3)	7.4 (1.2; 15.7)	25.0 (8.9; 41.1)	22.2 (5.5; 38.9)	0.358
Serum albumin deficiency < 35 g/L ^c^	5.0 (0.8; 8.8)	10.0 (0,5; 19.5)	37.0 (17.5; 56.5)	15.0 (7.2; 22.8)	**0.006**
**Dominating symptoms** ‒ % (%) Gastrointestinal manifestation	0.0 (0.0; 2.4)	8.1 (2.7; 13.5)	16.2 (9.2; 23.2)	18.9 (9.0; 28.9)	**0.004**
Respiratory manifestation	0.0 (0.0; 0.0)	0.0 (0.0; 2.4)	5.0 (1.5; 8.5)	11.8 (4.7; 18.9)	
Skin/mucosal manifestation	0.0 (0.0; 4.4)	0.0 (0.0; 1.8)	22.2 (10.9; 33.5)	11.1 (0.8; 21.4)	
**Diagnostic methods** Skin Prick Tests	0.0 (0.0; 2.4)	3.3 (0.8; 5.8)	10.0 (3.8; 16.2)	16.7 (9.6; 23.8)	0.012
Serum tests	0.0 (0.0; 6.4)	3.2 (0.6; 5.8)	9.7 (5.3; 14.1)	6.5 (2.4; 10.6)	
Oral challenge	0.0 (0.0; 0.0)	14.3 (2.2; 28.8)	42.9 (11.9;73.7)	28.6 (6.9; 50.3)	
**Therapy** Antihistamine drugs	4.4 (1.3; 7.5)	6.7 (3.1; 10.3)	24.4 (14.9; 33.8)	22.2 (9.9; 34.6)	**0.003**
Steroids	0.0 (0.0; 0.0)	4.8 (0.4; 9.5)	33.3 (11.3; 55.3)	23.8 (3.9; 43.6)	
Desensitization	0.0 (0.0; 0.0)	0.0 (0.0; 2.4)	33.3 (14.8; 51.9)	41.7 (21.2; 62.2)	
ELD	4.2 (1.5; 7.9)	10.1 (2.2; 18.0)	35.4 (21.7; 49.1)	14.6 (4.5; 24.7)	
**Additional dieting**	7.7 (3.0; 12.4)	3.8 (0.8; 7.0)	11.5 (8.3; 15.0)	19.2 (11.1; 27.4)	0.221
**Diet implementation** Medically consulted	7.5 (0.5; 14.5)	0.0 (0.0; 3.6)	9.4 (4.3; 14.5)	7.5 (2.1; 12.9)	**0.001**
Medically unconsulted	9.4 (1.7; 17.1)	13.2 (4.1; 22.3)	32.1 (19.8; 44.4)	26.4 (14.8; 38.0)	

FA^-^–control group, FA^+^S–allergy to single food-origin allergen, FA^+^P–polyallergy to various food-origin allergens, FA^+^M–mixed polyallergy to aero- and food-origin allergens. ELD–elimination diet. Data are presented as percentages with 95% confidence intervals. Significant associations are marked in bold.

^a^ *p* value of the Fisher’s exact test for comparison between groups with Bonferroni’s (*P*_B_) corrected significance threshold of 0.008.

^b^ Classified according to the WHO classification [21, 22].

^c^ Assessment or nutritional status [28].

#### Birth method and feeding type associations

The monitored population was homogeneous in terms of birth method. No differences were observed in the ED^+^ frequency according to the birth method. Significant differences were noted in the declared new-born feeding type **(S2 Table)** and its relationship with the health status and tendency towards eating abnormalities in both phases of the study (p<0.008), with a strong correlation of the features (r = 0.5983, *p =* 0.005). Exclusive breastfeeding was significantly more frequent in the control group than in each allergic group, and eating abnormalities were reported in less than 10% of these participants **(Table 2)**. In the FA^+^S and FA^+^P groups, mixed feeding co-occurred with a nearly 50% lower prevalence of ED and eating habits.

#### The place of residence, family history of allergy and its manifestation associations

The place of residence had no significant effect on the ED occurrence. Simultaneously, the initial moderate correlation (r = 0.498, *p =* 0.005) between living in the city and the tendency to eating abnormalities ceased to be no longer significant **(S2 Table)**.

A weak but significant increase (*p =* 0.024) in the number of people with positive family history of allergy in the allergic groups, with no implication on ED prevalence, was observed during the 5-year follow-up. Changes in the observed dominant symptoms with an increased severity of respiratory symptoms (*p =* 0.011) and skin/mucosal manifestations (*p =* 0.029), especially in the group of patients suffering from FA^+^P, were observed during follow-up studies. The type of manifestation was invariably moderately correlated with the tendency towards eating abnormalities (r = 0.5012; *p =* 0.005).

#### Applied medical procedures associations

The evaluation of applied medical procedures in allergic groups demonstrated that the dominant diagnostic method in the recruitment phase of the study was medical observation and skin prick tests, which were indicated with equal frequency in the FA^+^P and FA^+^M groups. A significant increase (*p =* 0.002) in the proportion of serum tests as a diagnostic method was observed in the follow-up phase **(Table 1)**. Regardless of the used diagnostic method and the diagnosed type of allergy, the predominant treatment approach was the implementation of an ELD that was followed by 65% and 74% of patients in the recruitment phase and the five-year follow-up phases, respectively. During the entire study period, there was a strong correlation between the predisposition to development of eating abnormalities and the type of applied therapeutic method (r = 0.7069; *p =* 0.005) **(Table 2)**. In addition, patients and their caregivers confirmed the implementation of additional non-medical diets, and only 15% of the participants on any type of diet confirmed that they had received nutrition counselling by a trained dietician or a GP before diet implementation, no matter how many products needed to be eliminated. In the FA^+^ group, self-implemented diet was very strongly correlated with ED (r = 0.8012; *p =* 0.005) and with reduced body weight parameters (r = 0.724; 0.005).

### Clinical outcomes of food allergy and eating disorders

#### Body weight status

Evaluation of weight revealed that over 30% of allergic children were classified as moderately underweight in the recruitment phase of the study, and this percentage has increased to over 40% in 5-year follow-up, whereas in the control group, this index did not exceed 15% (**Table 1**). There was also a greater proportion of children with low height for age in the recruitment phase of the study (17.7%) in FA^+^ group compared to that of their healthy peers (5.8%); however, this disproportion later became irrelevant. Less than 10% of the children were classified as overweight in the control group, and depending on the type of FA, this index ranged initially from 11% in the FA^+^S group to 23.6% in the FA^+^M group and from 0% in the FA^+^S group to 31.4% in the FA^+^P group in the follow-up phase.

Body weight status was partly correlated with the tendency towards eating abnormalities development (r = 0.6992; *p =* 0.005) regardless of the type of FA (**Table 2**). Twenty-five percent of the FA^+^ population in the recruitment phase of the study had W/H *z*-scores < -1.95, and W/A *z*-scores < -2.12 (**Table 3**). Moreover, after five years, a significant drop in these thresholds to <-2.25 and to < -2.20, respectively, was observed. For comparison, in the control group, this threshold was initially W/H *z*-score < -0.46 and W/A *z*-score < -0.98 and finally W/H *z*-score < -0.86 and W/A *z*-score < -1.17. In the FA^+^P group, a decrease in body weight during the 5-year period was the most significant (*p* = 0.0017), and the lowest *z*-scores were observed (W/A _*f*_ = -4.60; W/H _*f*_ = -4.20). The most significant increase in body weight was recorded for FA^+^M patients who were treated with inhaled steroids– 25% of the children reached *z*-scores W/A _*f*_ >2.55 and W/H _*f*_ >2.78 and 5.9% of the children met the criteria of obesity.

**Table 3:** Nutritional status of subjects and clinical parameters (allergic and inflammatory markers) in men and women by health status categories.

	Control Group (FA^-^)	Food allergy (FA^+^)					
		FA^+^S	*P-*value ^b^	FA^+^P	*P-*value ^b^	FA^+^M	*P-*value ^b^
**Female (*n* = 79)** *z-*score (W/H) _*r*_ ^a^	0.21 (-0.57–0.88)	-0.37 (-1.65–1.34)	0.286	-0.43 (-2.50–1.38)	0.071	0.49 (-1.36–1.79)	0.805
*z-*score (W/H) _*f*_ ^a^	0.12 (-0.78–0.99)	-0.78 (-2.19–2.18)	**0.016**	-0.33 (-2.50–1.43)	0.222	-0.55 (-2.61–3.06)	0.189
	*p =* 0.839 ^c^	*p =* 0.632 ^c^		*p =* 0.978 ^c^		*p =* **0.032** ^c^	
*z-*score (W/A) _*r*_ ^a^	0.07 (-1.01–1.30)	-0.66 (-1.12–0.18)	0.278	-0.68 (-2.39–1.02)	0.149	0.11 (-2.84–1.82)	0.912
*z-*score (W/A) _*f*_ ^a^	0.10 (-1.00–0.80)	-0.42 (-2.35–1.06)	**0.024**	-0.49 (-2.82–2.35)	**0.015**	-0.51 (-2.43–2.69)	**0.023**
	*p =* 0.723	*p =* 0.940 ^c^		*p =* 0.607 ^c^		*p =* 0.061 ^c^	
Total-IgE _*r*_ -kUA/L	26.31 [14.4]	521.8 [398.3]	0.281	223.9 [190.7]	0.326	709.2 [535.3]	**<0.001**
Total-IgE _*f*_ -kUA/L	33.1 [16.6]	840.9 [578.8]	**0.013**	1206.4 [486.8]	**<0.001**	922.2 [221.1]	**0.001**
	*p =* 0.725 ^c^	*p =* 0.391 ^c^		*p =* **0.016** ^c^		*p =* 0.723 ^c^	
Total- IgG _*r*_*—*g/L	3.84 [1.4]	9.7 [6.3]	0.067	9.5 [3.0]	**0.011**	8.4 [4.6]	**0.021**
IL-2 _*r*_ -pg/mL	9.91 [1.2]	16.1 [2.2]	0.613	25.1 [7.2]	**0.001**	25.5 [11.7]	**<0.001**
IL-4 _*r*_ -pg/mL	9.77 [1.1]]	49.5 [7.5]	**<0.001**	41.3 [9.6]	**<0.001**	40.5 [7.7]	**<0.001**
INF-γ _*r*_ -pg/mL	12.42 [2.5]	15.4 [4.2]	0.928	29.5 [9.9]	**<0.001**	26.5 [8.4]	**0.002**
**Male (*n* = 77)** *z-*score (W/H) _*r*_ ^a^	0.24 (-0.35–0.84)	-0.65 (-1.17–1.27)	0.213	-0.32 (-2.20–0.64)	**0.001**	-0.25 (-2.83–1.61)	**0.006**
	*p* = 0.845 ^d^	*p* = 0.792 ^d^		*p* = 0.926 ^d^		*p* = **0.042** ^d^	
*z-*score (W/H) _*f*_ ^a^	0.16 (-0.95–1.52)	-0.76 (-1.76–1.15)	0.064	-0.41 (-2.60–1.00)	0.320	-0.13 (-1.87–2.51)	0.454
	*p* = 0.895 ^c^; *p* = 0.867 ^d^	*p =* 0.744 ^c^; *p* = 0.921 ^d^		*p =* 0.867 ^c^; *p* = 0.930 ^d^		*p =* 0.643 ^c^; *p* = 0.463 ^d^	
*z-*score (W/A) _*r*_ ^a^	0.11 (-0.95–1.30)	0.52 (-2.27–0.93)	0.376	-0.13 (-2.26–1.23)	0.432	-0.43 (-1.89–2.13)	**0.032**
	*p* = 0.840 ^d^	*p* = **0.049** ^d^		*p* = 0.576 ^d^		***p* = 0.049** ^d^	
*z-*score (W/A) _*f*_ ^a^	0.03 (-1.53–0.74)	0.35 (-0.71–0.80)	0.496	-0.46 (-2.53–1.99)	**0.031**	-0.08 (-2.37–2.42)	0.253
	*p* = 0.658 ^c^; *p* = 0.623 ^d^	*p =* 0.540 ^c^; *p* = 0.069 ^d^		*p =* 0.319 ^c^; *p* = 0.690 ^d^		*p =* 0.271 ^c^; *p* = 0.604 ^d^	
Total-IgE _*r*_ -kUA/L	20.51 [10.8]	116.5 [23.2]	0.999	528.6 [182.3]	0.296	893.4 [1189.4]	**0.011**
	*p* = 0.297 ^d^	*p =* 0.484 ^d^		*p =* 0.394 ^d^		*p =* 0.157 ^d^	
Total-IgE _*f*_ -kUA/L	31.21 [18.1]	151.9 [13.2]	0.999	400.4 [275.8]	0.326	1332.7 [644.1]	**<0.001**
	*p* = 0.762 ^c^; *p* = 0.587 ^d^	*p =* 0.536 ^c^; *p* = 0.587 ^d^		*p* = 0.512 ^c^; ***p =* 0.049** ^d^		*p* = 0.410 ^c^; *p* = **0.039** ^d^	
Total- IgG _*r*_*-* g/L	3.67 [1.4]	5.5 [1.1]	0.979	8.0 [2.6]	**<0.001**	10.1 [4.4]	**<0.001**
	*p* = 0.900 ^d^	*p =* 0.219 ^d^		*p =* 0.656 ^d^		*p =* 0.760 ^d^	
IL-2 _*r*_ -pg/mL	10.12 [1.4]	10.4 [1.7]	0.999	24.6 [7.6]	**<0.001**	25.1 [8.9]	**<0.001**
	*p* = 0.540 ^d^	*p =* 0.142 ^d^		*p =* 0.738 ^d^		*p =* 0.953 ^d^	
IL-4 _*r*_ -pg/mL	10.16 [1.1]	42.9 [9.8]	**<0.001**	42.4 [9.6]	**<0.001**	41.8 [8.2]	**<0.001**
	*p* = 0.414 ^d^	*p =* 0.961 ^d^		*p =* 0.902 ^d^		*p =* 0.702 ^d^	
INF-γ _*r*_ -pg/mL	11.6 [2.6]	10.9 [1.9]	0.999	27.8 [10.8]	**0.005**	28.3 [8.5]	**0.003**
	*p* = 0.743 ^d^	*p =* 0.536 ^d^		*p =* 0.708 ^d^		*p =* 0.779 ^d^	

FA-–control group, FA^+^S–allergy to single food-origin allergen, FA^+^P–polyallergy to various food-origin allergens), FA^+^M–mixed polyallergy to aero- and food-origin allergens. Data are presented as the median (IQR) or means [SD]. Significant associations are marked in bold

^a^ *z*-scores calculated for weight/height (W/H) and for weight/age (W/A) [21, 22]

^b^ *p* value of the ANOVA-Tukey test for unequal sample sizes

^c^ *p* value of the Friedman test for dependent groups in follow-up studies

^d^ *p-*value of the t-test for the comparison male *vs*. female

_*r*_ Recruitment phase values

*f* Follow-up phase value

There was no general regularity detected in the relationship between the gender and *z*-scores value; however, female patients suffering from FA^+^M in the recruitment phase of study demonstrated weak but significantly higher and more accurate *z*-scores than those of the male participants (W/A: *p* = 0.042; W/H: *p* = 0.049). During the five years of the study, the average weight of female participants in the FA^+^M group decreased, and the differences between sex became irrelevant, despite the fact that in this group, most of the obese cases were reported.

#### Immunological parameters

Strong significant differences in analysed immunological parameters were determined for total IgE concentration not only between particular FA^+^ groups and the controls but also regarding the sex of the patients. Higher levels of IgE were observed in male groups, especially in the recruitment phase of the study, whereas in the follow-up phase, the differences were dependent on the type of manifested FA. The prevalence of ED was positively correlated with the total serum IgE concentration, but only in the female group (r = 0.5995; *p* = 0.005).

In the serum of majority of the FA^+^/ED^+^ patients, specific E antibodies against soybean (fl4), egg white (fl), cows’ milk (f2) and peanut (fl3) were detected **(S1 Fig and S2 Table)**. These combinations indicate that implementation of an elimination diet as a preventive method would require eliminating, in almost 52% of FA^+^ cases, two, and in 39% of the cases, three or more sources of allergens. The highest prevalence of ED was observed in the FA^+^P group following a multiple product elimination diet that excluded soybean (fl4), milk (f2) and wheat (f4) (24.8% prevalence of ED). In the FA^+^M group, the highest prevalence of ED (17.6%) was observed for a group of individuals allergic to egg white (fl), milk (f2), peanut (fl3), wheat (f4) and birch (t3). In the FA^+^S group, the highest prevalence of ED was observed in the group of patients suffering from an allergy to cows’ milk protein (16.7%).

Moreover, significant differences in serum content of total IgG, IL-2, IL-4 and INF-γ were observed; however, they were dependent only on the type of manifested FA and not on gender. These parameters were also related to the frequency of ED. IL-4/ INF-γ and IL-4/IL-2 ratios ranged from 1.42 to 4.13, which confirms that the immune response of FA^+^ patients is driven by a humoral and not a pure inflammatory mechanism **(Table 3)**.

The secretion of analysed cytokines was elevated despite 33.8% of the allergic patients was diagnosed as suffering from lymphopenia and implement ELD. The reduced absolute number of lymphocytes in the blood and lower concentration of albumin were most frequently observed in FA^+^P patients following multiple product ELD (22.4%, and 37.8%, respectively) in comparison to levels observed in their healthy peers (5.3% and 6.7%, respectively), FA^+^S patients (16.7% and 33.3%, respectively) and FA^+^M patients (14.3% and 20%, respectively). Despite observed differences in levels of both blood parameters among FA^-^ and FA^+^ groups, their possible co-occurrence with ED was observed only for reduced levels of albumin, which was a moderately negative correlation (r = -0.499; *p =* 0.005) **(Table 2).**

### Correlations of eating disorders in food allergy

Results of a multivariate logistic regression model demonstrated that three combined factors had the strongest influence on ED prevalence in the analysed population: used allergy diagnostic method, dominant allergy symptoms, and therapy implementation (Adj.2), especially in underweight, polyallergic groups **(Table 4).** Odds ratios of ED in most of the allergic groups with weight abnormalities were higher (OR>1) than those in the control group. Analysis of probability of ED occurrence in the studied groups adjusted to new-born feeding type, parents’ reported food allergy and residence (Adj.1) indicated a greater sensitivity of female participants (ORs >1.23) than male, where most of the male group ORs were below the values for females. The strongest association of ED prevalence and combination of both features (Adj.1 and Adj.2) was observed in the FA^+^P group.

**Table 4:** Odds ratios (OR) for ED prevalence in particular food allergy groups of males and females, compared to the correct weight category children adjusted for Adj.1: new-born feeding type, parent-reported food allergy, residence and Adj.2: Used allergy diagnostic method, dominant allergy symptoms, and therapy implementation.

Prevalence of ED^+^ in particular food allergy (FA^+^) group	*z*-score W/H	Male						Female					
		OR Adj.1	95% CI	p (LR) ^a^	OR Adj.2	95% CI	p (LR) ^a^	OR Adj.1	95% CI	p (LR) ^a^	OR Adj.2	95% CI	p (LR) ^a^
FA^+^S	≤ -2	0.81	0.40–1.62	0.551	0.90	0.31–2.62	0.847	1.99	0.88–4.52	0.101	1.91	1.05–3.45	**0.047**
	≥ +2	1.17	0.45–3.02	0.744	1.25	0.55–2.85	0.590	1.23	0.62–2.42	0.557	1.22	0.61–2.44	0.572
FA^+^P	≤ -2	0.92	0.43–1.97	0.836	2.77	1.07–4.16	**0.034**	1.99	1.15–3.23	**0.015**	3.17	3.17–8.19	**0.017**
	≥ +2	1.29	1.14–2.62	**0.013**	1.46	1.06–1.90	**0.036**	1.37	1.17–1.83	**0.014**	1.05	0.73–1.48	0.848
FA^+^M	≤ -2	1.014	0.90–1.14	0.810	2.24	1.44–3.62	**0.028**	1.50	0.62–3.64	0.370	1.18	1.06–1.57	**0.004**
	≥ +2	1.63	0.73–3.62	0.235	1.57	1.23–2.38	**0.021**	1.47	0.65–3.30	0.381	0.86	0.39–1.90	0.704

FA^+^S–allergy to single food-origin allergen, FA^+^P–polyallergy to various food-origin allergens), FA^+^M–mixed polyallergy to aero- and food-origin allergens. Significant associations are marked in bold.

^a^ *p* (LR) value of the Wald likelihood ratio test (LR test) for significance evaluation of the model.

**Fig 1:**
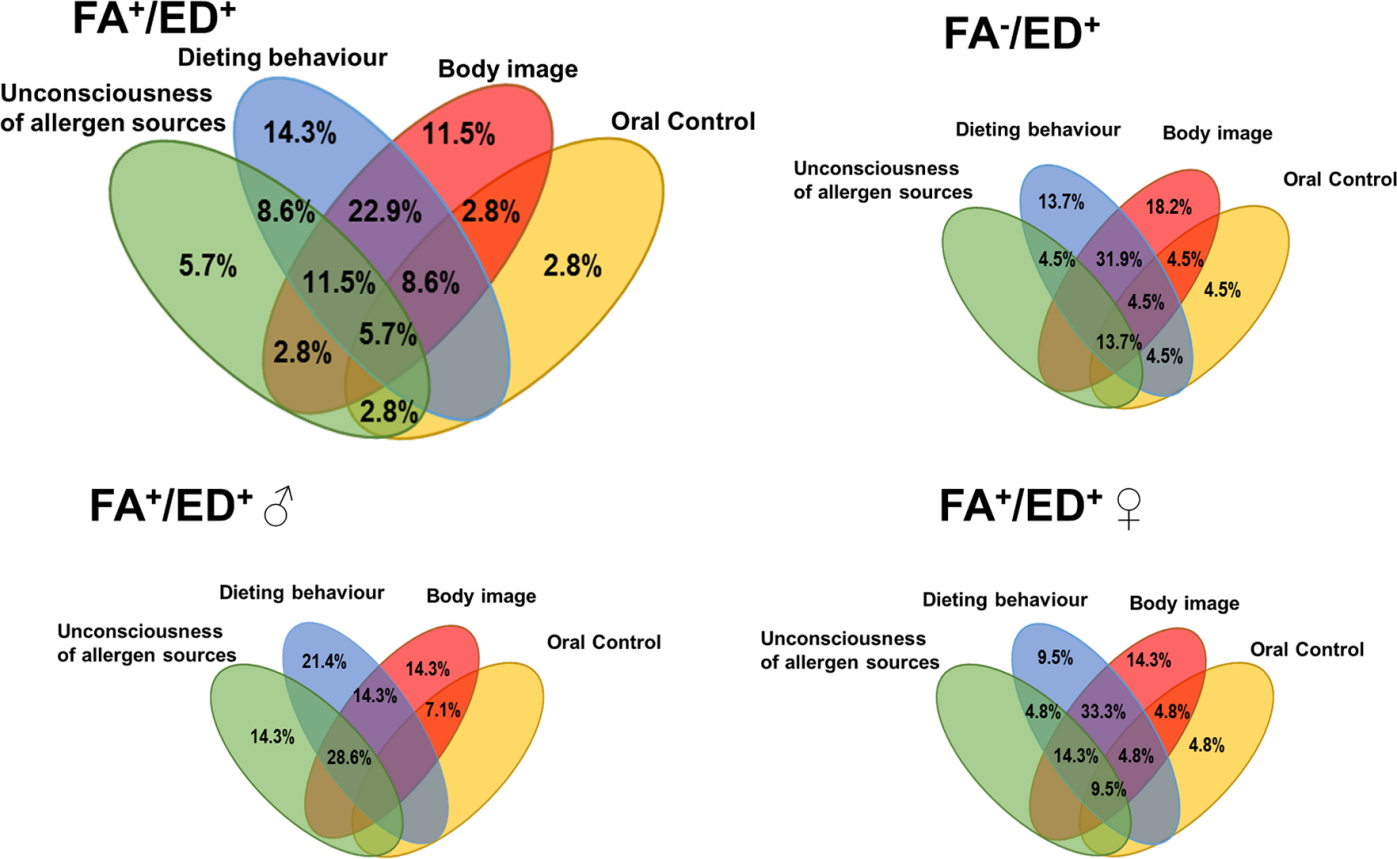
Allergic problem awareness and eating attitude factors in the FA^+^/ED^+^ groups with body weight abnormalities. Parameters such as oral control, dieting behaviour and body image were evaluated based on affirmative answers to the questions in the ED tests and the additional questions in the form for assessing the psychosocial status of patients with allergies. Insufficient allergy problem awareness was reported based on incorrect answers on at least five questions devoted to allergy problems.

Considering questions about behavioural and allergic awareness in the ED screening test, in both the FA^+^/ED^+^ and FA^-^/ED^+^ groups, supreme over-concern about body weight and dieting behaviour was reported **(Fig 1)**. Body image dissatisfaction was the dominant factor in both non-allergic group and female representatives of allergy sufferers. In the male FA^+^/ED^+^ group, the dominant factors turned out to be dieting behaviour co-occurring with unconsciousness of allergen sources and lack of confidence in allergy issue (28.6%). Increased oral control behaviours were reported in the female FA^+^M group (23.9%).

## Discussion

### Growth abnormalities and dysfunction of immune system in FA

#### Prevalence of FA^+^ED^+^ and body weight status

To our knowledge, this is the first follow-up study establishing ED occurrence concomitantly with persistent growth abnormalities and dysfunction of the immune system in children with clinically diagnosed FA. The present study covered the north-eastern Polish population living in the area of Green Lungs of Europe, which may explain the low prevalence of FA at 2%. In comparison in the central, rural part of Poland, medically confirmed FA was reported at approximately 8% [6]. In the European population FA prevalence, estimated based on meta-analysis, range from 0.1 to 6.0% [29].

The prevalence may vary slightly between regions and countries; nevertheless, the frequency of declared essential medical procedures applied in FA remains the same, with a dominant role of ELD. This procedure was also the most popular in our study. Moreover ELD has been observed to be correlated with an increased risk of underweight cases, especially in the group excluding multiple products. This result was consistent with Meyer’s [30], who described the UK population, where malnutrition and underweight individuals formed 8.5% of the allergic population, as well as with Flammarion’s [31], who reported the percentage of such cases in the French population at 9.3%. Nevertheless, the ratio of underweight individuals in our population reached almost 30%. This may be explained by three times less frequent dietician supervision compared to that observed in populations of the abovementioned reports. Most FA guidelines recommend including growth assessment for the timely prevention of poor growth, which is one of the indicative factors of malnutrition. Neglect by caregivers of obligatory control after partial relief of symptoms and lack of confidence in the seriousness of the illness may explain the stronger disproportion in the analysed population.

Also the education of young patients suffering from chronic allergy, about allergen sources and procedures for dealing with severe allergic reactions, seems to be neglected; 35.4% of the patients and 18.7% of caregivers reveal a lack of confidence in this issue. This may explain the reason for a strong and prolonged association of FA and BMI *z*-score disproportion observed in the analysed FA^+^ population (approximately 30% of the underweight individuals and >30% of the overweight individuals, respectively). It was more significant than that reported in the previously described Italian and Finnish populations suffering from other atopic diseases, such as wheezing (6% / 10%), allergic rhinitis (21% / 26%), asthma (3.5% / 9%) and atopic dermatitis (16% / 20%) [32, 33]. In such diseases, an elimination is also often implemented because part of the food-delivered antigens, such as those from eggs or milk, aggravate their symptoms.

#### The impact of applied medical procedures on immunological parameters of FA^+^ED^+^ patients

An increased prevalence of overweight individuals in FA^+^ED^+^ patients could be associated with side effects of steroid therapy, reduced physical activity, and severity of respiratory symptoms in children [34]. Also incorrect replacement of dietary products containing allergenic proteins with products containing higher carbohydrate and lower protein content in the diet may be the reason of the observed overweight. This situation was greatly strengthened in our study, where lower albumin content, especially in the group with polyallergy to various foods following multi-product ELD, was observed, which could be explained by following the diet long-term, with reduced protein supply (albumin t½ = 20 days).

One explanation of decreased concentration of serum albumin could be the loss of nutrients caused by abnormally increased intestinal permeability, despite the implemented elimination diet, which is often observed in children suffering from cows’ milk protein allergy (CMPA). In severe cases, even trace amounts of antigen remnants may cause an increase in the leaking of the intestine [35]. In our studied population, over 50% of the participants declared prophylactic, sometimes without medical indication, use of infant hydrolysed formulas instead of breastfeeding. It has been done even if the infant was not diagnosed as suffering from CMPA. This procedure was common even though it has been stated that formula feeding may be responsible for low body weight and weaker weight-gain of new-borns. In the context of the studies showing that children suffering from atopic disease may have higher caloric and protein needs, primarily if the dominant symptoms of the disease come from skin and mucus [36,31] such procedure can lead to underweight. In our study, it was observed that this type of manifestation was almost as frequent as gastrointestinal manifestations.

As a result of the use of various dietary treatments, a decreased level of albumin in our population was observed, which may also be observed during inflammatory diseases of the intestine [37], infections and in other states [38]. This may confound the interpretation of results; however, in our study, we excluded cases with severe inflammatory diseases in the recruitment phase.

#### The impact of gender and intestinal mucosal integrity on immunological parameters of FA^+^ED^+^ patients

Moreover, additional factors, such as lymphopenia evaluation and BMI-based factors, were taken into account according to the guidelines of the European Society for Clinical Nutrition and Metabolism [28]. As a result, it was stated that despite the dominant humoral, IgE-mediated mechanism of FA, the frequency of lymphopenia was surprisingly high for FA patients, which was also significantly correlated with the multiple product elimination, both in obese and in underweight FA patients. Despite the occurrence of lymphopenia, we confirmed a typical for allergy Th1/Th2 imbalance with a predominance of the Th2-type response. This imbalance was related to the type of allergy and dominant symptoms but also to the BMI-*z*-scores and gender. It was analogous to the results obtained by Kilpeläinen et al. [32], which were focused on the evaluation of atopy prevalence in Finnish children with varying BMI-*z*-scores and levels of physical activity.

In overweight/obese individuals and in female subjects, it was explained by an adipokine-dependent mechanism of decreased immunological tolerance to allergens previously described in terms of asthma and allergic rhinitis [39, 40]. Although, it has not been linked to FA so far, it is known that leptin, as the satiety hormone produced by adipocytes, wherein its concentration can be increased in obese people. Also in females patients it may induce a Th1 response. In overweight patients, leptin-resistance may cause locally enhanced production Th1 cytokines such as INF-γ and TNF-α in adipocyte tissue, but it also stimulates IL-6 expression in stimulated macrophages, which in turn increases production of IL-4 and IL-5 systemically under antigen exposure [39,41,42]. The observed systemic increase in IL-4 concentration may be explained by the decrease in immunological tolerance to even trace amounts of allergens as a consequence of immunological changes induced by leptin resistance. This observation is analogous to the study of van Huisstede et al. [43], who evaluated the presence of systemic inflammation as an effect of obesity in asthma.

On the other hand, observed lymphopenia with constant, strong Th2-mediated responses in underweight participants may be explained by the effects of impairment of intestinal mucosal integrity, as well as by imbalanced microbiota composition. It has been demonstrated by Blanton et al., [44] in a Malawian population that immaturity of intestinal microbiota and the resultant metabolic abnormalities may causally be related to underweight status in children. It was also confirmed that immature microbiota from undernourished infants/children may transmit impaired growth phenotypes, which was verified in male germ-free mice models. There are also reports of disturbances in the structure of gut microbiota in underweight patients suffering from ED that reveal an increase in methanogens in the intestinal population [45] or decrease in *Lactobacillus reuteri* [46]. Similar regularities in microbiota disproportions were previously observed in the course of FA by other studies [47, 48]. It has been associated with an impairment of the microbial PAMP/TLR interactions, enhanced Th2 responses towards antigens and consequent exacerbation of the condition of patients. To sum up this part of the study, we conclude that incorrectly composed elimination diet may directly or indirectly affect immune system activity in FA by causing body weight abnormalities, resulting in impairment of cell-mediated immunity, cytokine production, mucosal permeability, antibody affinity [20] and possibly gut microbial dysbiosis.

### ED and psychosocial implications of FA

#### Psychosocial implications in FA^+^ED^+^ development

ED and psychosocial implications manifested by increased sensitivity to own appearance typically emerge during adolescence and adulthood but increasing number of cases have been reported even in elementary school and according to the author’s observations, such cases may co-occur with FA. Such a phenomenon was previously suggested by Shanahan et al. [49], who analysed a population of children in the US (North Carolina state). The association of underweight individuals and disturbed body image, classified as *anorexia nervosa*, with symptoms of FA in that population reached 7.8%. In the currently studied north-eastern Polish population, we observed a stronger association between FA and ED (24.8%); however, unlike Shanahan’s study, overweight children were also included in our study because of frequently confirmed compulsive behaviours. Stronger association of FA and ED may have been caused due to a greater homogeneity of the analysed population of allergy sufferers included by clinicians, unlike the population in the abovementioned study, where FA was parent-reported and not physician-verified. Such studies, however, allow for the assessment of the impact of a greater number of psychosocial disorders that may co-exist in the course of FA. For example, Patten et al. [50] observed that FA was significantly associated with mood and anxiety disorders and OR for depression was 1.8 (95% CI 1.5–2.3). Associations of comparable strength were observed for bipolar disorder and panic disorders. Other scientists described several mental abnormalities directly associated with the process of eating as further repercussions of FA in early childhood.

#### Behavioural implications and impact of lack of confidence in FA issues on FA^+^ED^+^ development

A common problem that remains among children is selective eating, which consists of eating a very limited range of foods, especially of a particular colour, texture, or brand, with excessive preoccupation with eating healthy food [51]. Furthermore, food phobias can be additionally enhanced by fear of choking, swallowing or vomiting likely preceded by traumatic experiences occurring e.g., in the course of FA [52]. In the studied population, the origins of selective eating and *orthorexia nervosa* were observed especially in male patients. In female participants dominating ED, there were characteristic of *bulimia nervosa* in the overweight and *anorexia nervosa* in the underweight group. It should be stressed that ED was strongly associated with applied therapeutic methods regardless of gender. In the studied population, an increased body weight after steroid therapy and decreased body weight after common, seasonal antihistamine over-dosage was associated with the prevalence of ED. Explanation for this phenomenon could be alterations in histamine signalling in the brain implicated in the inverse relationship between histamine activity and food intake, which in turn results in malnutrition and increases susceptibility to the development of depressive states and *anorexia nervosa* [53]. Another possible explanation of elevated psychosocial implications may reflect syndromes of adaptations to living with FA. Living with food allergies poses a unique stress for children, adolescents and their parents, resulting in disruptions of daily life [4], and burdens the activities of adolescents that are crucial for proper psychosocial development in relation to peers [54]. The most aggravating factors in cited studies were attempts to strictly follow an elimination diet, which were also observed in our study. Similar results were observed in the population of children (n = 20) suffering from peanut allergy that demonstrated more impaired quality of life and lower body image confidence than those suffering from insulin-dependent diabetes mellitus (n = 20). In that population, the children suffering from FA reported more fear of an adverse event, more anxiety about eating, and more restriction due to the illness [55].

Finally, there is one possible explanation of strong association of ED and FA that is focused on cognitive-emotional sensitization that involves the central nervous system in complex neuronal networks. Extensive activation of these cognitive networks (prolonged stress), sometimes triggered by peripheral mechanisms (inconvenient FA manifestation and local inflammation), might be a crucial mechanism underlying several subjective health complaints and may lead to a greater perception of illness, deterioration of health and loss of weight [56]. This was originally confirmed by Bell et al. [57] in a group of 490 participants who reported sensitivity to food (wheat, dairy, eggs) and chemicals (pesticide, car exhaust, paint, perfume, new carpet). Although higher emotional deterioration and stronger FA symptoms coexisting with depression symptoms and body weight faltering were observed in these patients, these symptoms were mostly correlated with somatization not with strict immunologic reaction. Based on the aforementioned studies, it could be concluded that the mental condition of patients is an important element in the course of both allergic diseases and ED.

### Limitations and directions for future research

First, the population of the north-eastern region of Poland is representative of the communities from which it was sampled, but probably not of the whole Polish population. Findings should be extended and confirmed in studies that include participants from other regions. Nevertheless, the Polish population is perceived to be rather homogeneous in ethnic and racial terms. Second, the population of FA children were recruited based on serum tests and symptomatic evaluation of clinicians, and not based on double-blind food challenges, which is seen as a reference method in the diagnosis of FA [58]. This may be perceived as a limitation of the present study; however, we wanted to establish the psychosocial impact of different variables on ED frequency, taking into consideration the most common and acceptable allergy diagnostic criteria used in Poland.

Third, the age of children in the follow-up phase ranged from 5–18 years and required double self- and parent-reported ED evaluation because almost 9% of the children were too young to cope on their own with the SCOFF and EAT-8 questionnaire, which may cause slight overestimation of the prevalence of ED. On the other hand, the youngest children unable to respond on the questionnaire on their own have been categorized into various FA groups including control, so the general trend is not falsified. Finally, the present study is limited by no intestinal microbiota biodiversity information of the studied population. However, a case study report about observed disproportion of skin microbiota in female representative of studied population has been previously published [59]. Nonetheless, the study provides a strong background for future research devoted to microbiota. Moreover, while the investigations should be continued on present group of participants, the pool of subjects should be expanded to include groups of adult and elderly people with FA and people who have been diagnosed as underweight or with confirmed *anorexia nervosa*, who during their childhood suffered from allergy but in whom tolerance has developed.

## Conclusions

To recapitulate, this work confirms the correlation between the state of allergy, applied procedures, weight abnormalities and ED occurrence in early life. Proper allergy education and dietician-supervised establishment of diet, especially for people with a positive family history of allergy, may contribute to more efficient allergy therapy in early life and in the reduction of ED in the future. An important factor seems to be the different psychosocial susceptibilities of allergic people, whereby evaluation of such susceptibilities could be included in the assessment of the health condition of the FA patients. Studies on the impact of ED and psychosocial implications on intestinal microbiota and immune system in FA but also in the course of other diseases should be continued due to the need of a pathomechanism explaining the conditions and to enlarge the scale of the conducted research.

## Supporting information

S1 Table Inclusion-exclusion criteria and specified assessments. (DOCX)

S2 Table Comprehensive characteristics of the studied underage population. (DOCX)

S1 Text Characteristic of the ED prevalence tests. (DOCX)

S1 Fig Summary of the most common food allergies and mixed polyallergies determined in characterized FA+/ED+ groups. (TIF)

## Abbreviations

AD: anti-allergic drugs
ED: eating disorders
FA: food allergy
ELD: elimination diet
FD: feeding disorders
FA^+^/ED^-^: the prevalence of allergy to food in studied population
FA^-^/ED^+^: the prevalence of disordered eating in studied population
FA^+^/ED^+^: the prevalence of combined factors (allergy to food & disordered eating)
FA^-^/ED^-^: children without respective symptoms in studied population
FA^+^S: allergy to a single food origin allergen
FA^+^P: polyallergy to various food allergens
FA^+^M: mixed polyallergy to aero- and food allergens
R: recruitment phase values
F: follow-up phase values
IgE: immunoglobulins of class E
*s*IgE: specific to food immunoglobulins of class E
IL-2: Interleukin 2
IL-4: Interleukin 4
INF-*γ*##VWW##: Interferon gamma
ELISA: enzyme linked immunosorbent assay
BMI *z*-scores: Body Mass Index *z*–scores
W/A *z*-score: Body Mass Index *z*-scores adjusted to weight and age
W/H *z*-score: Body Mass Index *z*-scores adjusted to weight and height
H/A *z*-score: Body Mass Index *z*-scores adjusted to height and age
EAT-8: The Eating Attitudes Test
SCOFF: screening test of eating disorders
IQR: interquartile range
ORs: Odd ratios
SD: standard deviation
PB: Bonferroni’s corrected significance threshold
LR: the Wald likelihood ratio test for significance evaluation of the model

## References

[1] Pawankar R, Holgate ST, Canonica GW, Lockey RF. White Book on Allergy Update World Allergy Organization: Milwaukee Press; 2013.

[2] Fiocchi A, Bahna SL, Berg A Von, Beyer K, Bozzola M, Compalati E, et al World Allergy Organization (WAO) Diagnosis and Rationale for Action against Cow Õ s Milk Allergy (DRACMA) Guidelines. 2010;21:1–125. 10.1111/j.1399-3038.2010.01068.x 20618740

[3] Young E, Stoneham MD, Petruckevitch A, Barton J RR. A population study of food intolerance. Lancet. 1994;343(8906):1127–30. 7910231 10.1016/s0140-6736(94)90234-8

[4] Teufel M, Biedermann T, Rapps N, Hausteiner C, Henningsen P, Enck P, et al Psychological burden of food allergy. World J Gastroenterol. 2007; https://doi.org/10.3748/wjg.v13.i25.3456 10.3748/wjg.v13.i25.3456 PMC4146781 17659692

[5] Okada Y, Yamashita T, Kumagai H, Morikawa Y, Akasawa A. Accurate determination of childhood food allergy prevalence and correction of unnecessary avoidance. Allergy, Asthma Immunol Res. 2017;9(4):322–8. 28497919 10.4168/aair.2017.9.4.322 PMC5446947

[6] Stoma M, Slaska-Grzywna B, Zukiewicz-Sobczak WA, Kostecka M, Bojanowska M, Dudziak A, et al Food allergies in rural areas. Postep dermatologii i Alergol. 2016;33(4):281–5. 10.5114/ada.2016.61604 PMC5004217 27605899

[7] Kosky, Nick SM, Lacey JH. Bulimia nervosa and food allergy: A case report. Int J Eat Disord. 1993;14(1):117–9. 8339094 10.1002/1098-108x(199307)14:1<117::aid-eat2260140116>3.0.co;2-h

[8] Ricca V, Mannucci E, Calabrò A, Bernardo MDi, Cabras PL, Rotella CM. Anorexia Nervosa and Celiac Disease: Two Case Reports. Int J Eat Disord. 2000; 27: 119–22. 10590459 10.1002/(sici)1098-108x(200001)27:1<119::aid-eat16>3.0.co;2-r

[9] Yucel B, Ozbey N, Demir K, Polat A, Yager J. Eating Disorders and Celiac Disease: A Case Report. Int J Eat Disord. 2006;39:530–2. doi: 10.1002/eat.20294 16715485 10.1002/eat.20294

[10] Wilczynska-Kwiatek A, Bargiel-Matusiewicz K, Lapinski L. Asthma, allergy, mood disorders, and nutrition. Eur J Med Res. 2009;14 Suppl 4:248–54. 20156766 10.1186/2047-783X-14-S4-248 PMC3521357

[11] Boyce JA, Assa’ad A, Burks AW, Jones SM, Sampson HA, Wood RA, et al Guidelines for the Diagnosis and Management of Food Allergy in the United States: Report of the NIAID-Sponsored Expert Panel. J Allergy Clin Immunol. 2010;126(6 0):S1–58. 21134576 10.1016/j.jaci.2010.10.007 PMC4241964

[12] Mollet A, Meier S, Riediger T, Lutz TA. Histamine H 1 receptors mediate the anorectic action of the pancreatic hormone amylin. Peptides. 2003;24:155–8. 12576097 10.1016/s0196-9781(02)00288-7

[13] Malmlöf K, Zaragoza F, Golozoubova V, Refsgaard H, Cremers T, Raun K, et al PAPER Influence of a selective histamine H 3 receptor antagonist on hypothalamic neural activity, food intake and body weight. Int J Obes. 2005;29:1402–12. 10.1038/sj.ijo.0803036 16151415

[14] Chung BY, Cho SI, Ahn IS, Lee HB, Kim HO, Park CW, et al Treatment of atopic dermatitis with a low-histamine diet. Ann Dermatol. 2011;23(SUPPL. 1):91–5. 10.5021/ad.2011.23.S1.S91 PMC3199434 22028584

[15] Kacik J, Wróblewska B, Lewicki S, Zdanowski R, Kalicki B. Serum Diamine Oxidase in Pseudoallergy in the Pediatric Population In Boston, MA: Springer US; 2017 p. 1–10. 10.1007/5584_2017_81 28804811

[16] Ciriaco M, Ventrice P, Russo G, Scicchitano M, Mazzitello G, Scicchitano F, et al Corticosteroid-related central nervous system side effects. J Pharmacol Pharmacother. 2013;4(5):94. 10.4103/0976-500X.120975 PMC3853679 24347992

[17] Madden S, Morris A, Zurynski YA, Kohn M, Elliot EJ. Burden of eating disorders in 5-13-year-old children in Australia. Med J Aust. 2009;190(8):410–4. 19374611 10.5694/j.1326-5377.2009.tb02487.x

[18] Carper JL, Orlet Fisher J, Birch LL. Young girls’ emerging dietary restraint and disinhibition are related to parental control in child feeding. Appetite [Internet]. 2000;35(2):121–9. doi: 10.1006/appe.2000.0343 10986105 10.1006/appe.2000.0343

[19] Kurz S, Van Dyck Z, Dremmel D, Munsch S, Hilbert A. Variants of early-onset restrictive eating disturbances in middle childhood. Int J Eat Disord. 2016; https://doi.org/10.1002/eat.22461 10.1002/eat.22461 26356990

[20] Marcos A, Nova E, Montero A. Changes in the immune system are conditioned by nutrition. Eur J Clin Nutr. 2003;57:S66–9. doi: 10.1038/sj.ejcn.1601819 12947457 10.1038/sj.ejcn.1601819

[21] WHO Child Growth Standards: Length/height—for-age, weight-for-age, weight-for-length, weight-for-height and body mass index-for-age: Methods and development. WHO Multicentre Growth Reference Study Group. World Health Organization, Geneva Press; 2006.

[22] WHO Anthro V. 3.2.2. Retrieved from http://www.who.int/childgrowth/software/en/, 2011. Last accessed June 9, 2015.Wilczynska-Kwiatek, A., Bargiel-Matusiewicz, K., & Lapinski, L. (2009). Asthma, allergy, mood disorders, and nutrition. European Journal of Medical Research, 14 Suppl 4, 248–54. https://doi.org/10.1186/2047-783X-14-S4-248 10.1186/2047-783X-14-S4-248 PMC3521357 20156766

[23] WHO & UNICEF, The State of the World's Children, 2009. Moccia, P. ISBN: 978-92-806-4318-3

[24] Morgan JF, Reid F, Lacey J. H. The SCOFF questionnaire: Assessment of a new screening tool for eating disorders. Bmj. 1999;319:1467–8. 10582927 10.1136/bmj.319.7223.1467 PMC28290

[25] Richter F, Strauss B, Braehler E, Altmann U, Berger U. Eating Behaviors Psychometric properties of a short version of the Eating Attitudes Test (EAT-8) in a German representative sample. Eat Behav. 2016;21:198–204. doi: 10.1016/j.eatbeh.2016.03.006 26978119 10.1016/j.eatbeh.2016.03.006

[26] Hill LS, Reid F, Morgan JF, Lacey JH. SCOFF, the development of an eating disorder screening questionnaire. Int J Eat Disord. 2010;43(4):344–51. doi: 10.1002/eat.20679 19343793 10.1002/eat.20679

[27] Bächle C, Stahl-Pehe A, Rosenbauer J. Disordered eating and insulin restriction in youths receiving intensified insulin treatment: Results from a nationwide population-based study. Int J Eat Disord. 2016; https://doi.org/10.1002/eat.22463. 10.1002/eat.22463 26395028

[28] Ellegård L. Assessment or nutritional status–what are we measuring? Blood tests in the assessment of nutritional status -an overwiew. 33 ESPEN Congress Gothenburg 2011. http://www.espen.org/presfile/Ellegard_2011.pdf.

[29] Nwaru BI, Hickstein L, Panesar SS, Roberts G, Muraro A, Sheikh A. Prevalence of common food allergies in Europe: A systematic review and meta-analysis. Allergy Eur J Allergy Clin Immunol. 2014;69(8):992–1007. 10.1111/all.12423 24816523

[30] Meyer R, De Koker C, Dziubak R, Venter C, Dominguez-Ortega G, Cutts R, et al Malnutrition in children with food allergies in the UK. J Hum Nutr Diet. 2014;27(3):227–35. doi: 10.1111/jhn.12149 23937486 10.1111/jhn.12149

[31] Flammarion S, Santos C, Guimber D, Jouannic L, Thumerelle C, Gottrand F, et al Diet and nutritional status of children with food allergies. Pediatric Allergy and Immunology : Official Publication of the European Society of Pediatric Allergy and Immunology. 2011;22(2):161–5. 20561235 10.1111/j.1399-3038.2010.01028.x

[32] Kilpeläinen M, Terho EO, Helenius H, Koskenvuo M. Body mass index and physical activity in relation to asthma and atopic diseases in young adults. Respir Med. 2006; https://doi.org/10.1016/j.rmed.2006.01.011 10.1016/j.rmed.2006.01.011 16503404

[33] Ciprandi G, Pistorio A, Tosca M, Ferraro MR, Cirillo I. Body mass index, respiratory function and bronchial hyperreactivity in allergic rhinitis and asthma. Respir Med. 2009; https://doi.org/10.1016/j.rmed.2008.08.008 10.1016/j.rmed.2008.08.008 18818065

[34] Byberg KK, Eide GE, Forman MR, Júlíusson PB, Øymar K. Body mass index and physical activity in early childhood are associated with atopic sensitization, atopic dermatitis and asthma in later childhood. Clin Transl Allergy. 2016;6(1):33 29. doi: 10.1186/s13601-016-0124-9 27559467 10.1186/s13601-016-0124-9 PMC4995660

[35] Farhadi A, Banan A, Fields J, Keshavarzian A. Intestinal barrier: An interface between health and disease. J Gastroenterol Hepatol. 2003;18(5):479–97. 12702039 10.1046/j.1440-1746.2003.03032.x

[36] Isolauri E, Sütas Y, Mäkinen-Kiljunen S, Oja SS, Isosomppi R, Turjanmaa K. Efficacy and safety of hydrolyzed cow milk and amino acid-derived formulas in infants with cow milk allergy. J Pediatr. 1995;127(4):550–7. 7562275 10.1016/s0022-3476(95)70111-7

[37] Fortunato JE, Scheimann AO. Protein-Energy Malnutrition and Feeding Refusal Secondary to Food Allergies. Clin Pediatr (Phila). 2007; 5;47(5):496–9. 10.1177/0009922807310937 18509149

[38] Bharadwaj S, Ginoya S, Tandon P, Gohel TD, Guirguis J, Vallabh H, et al Malnutrition: laboratory markers vs nutritional assessment. Gastroenterol Rep. 2016; https://academic.oup.com/gastro/article-lookup/doi/10.1093/gastro/gow013 10.1093/gastro/gow013 PMC5193064 27174435

[39] Santamaria F, Montella S, De Stefano S, Sperlı F, Barbarano F, Spadaro R, et al Asthma, atopy, and airway inflammation in obese children. J Allergy Clin Immunol. 2007;120(4):965–7. doi: 10.1016/j.jaci.2007.06.002 17637474 10.1016/j.jaci.2007.06.002

[40] Sideleva O, Suratt BT, Black KE, Tharp WG, Pratley RE, Forgione P, et al Obesity and asthma: An inflammatory disease of adipose tissue not the airway. Am J Respir Crit Care Med. 2012;186(7):598–605. doi: 10.1164/rccm.201203-0573OC 22837379 10.1164/rccm.201203-0573OC PMC3480522

[41] McLachlan CR, Poulton R, Car G, Cowan J, Filsell S, Greene JM, et al Adiposity, asthma, and airway inflammation. J Allergy Clin Immunol. 2007;119(3):634–9. doi: 10.1016/j.jaci.2006.10.029 17141852 10.1016/j.jaci.2006.10.029

[42] Dourado LPA, Noviello M de LM, Alvarenga DM, Menezes Z, Perez DA, Batista NV, et al Experimental food allergy leads to adipose tissue inflammation, systemic metabolic alterations and weight loss in mice. Cell Immunol. 2011;270(2):198–206. doi: 10.1016/j.cellimm.2011.05.008 21636080 10.1016/j.cellimm.2011.05.008

[43] van Huisstede A, Rudolphus A, Castro Cabezas M, Biter LU, van de Geijn G-J, Taube C, et al Effect of bariatric surgery on asthma control, lung function and bronchial and systemic inflammation in morbidly obese subjects with asthma. Thorax. 2015; 12;70(7):659 LP–667. 25934136 10.1136/thoraxjnl-2014-206712

[44] Blanton L V, Charbonneau MR, Salih T, Barratt MJ, Ilkaveya O, Subramanian S, et al Gut bacteria that rescue growth impairments transmitted by immature microbiota from undernourished children Laura. Science (80-). 2016;351(6275):1–18. 10.1126/science.aad3311 PMC4787260 26912898

[45] Million M, Angelakis E, Maraninchi M, Henry M, Giorgi R, Valero R, et al Correlation between body mass index and gut concentrations of Lactobacillus reuteri, Bifidobacterium animalis, Methanobrevibacter smithii and Escherichia coli. Int J Obes. 2013;37(11):1460–6. 10.1038/ijo.2013.20 PMC3826031 23459324

[46] Armougom F, Henry M, Vialettes B, Raccah D, Raoult D. Monitoring bacterial community of human gut microbiota reveals an increase in Lactobacillus in obese patients and Methanogens in anorexic patients. PLoS One. 2009; https://doi.org/10.1371/journal.pone.0007125 10.1371/journal.pone.0007125 PMC2742902 19774074

[47] Sjögren YM, Jenmalm MC, Böttcher MF, Björkstén B, Sverremark-Ekström E. Altered early infant gut microbiota in children developing allergy up to 5 years of age. Clin Exp Allergy. 2009;39(4):518–26. doi: 10.1111/j.1365-2222.2008.03156.x 19220322 10.1111/j.1365-2222.2008.03156.x

[48] Klewicka E, Cukrowska B, Libudzisz Z, Ślizewska K, Motyl I. Changes in gut microbiota in children with atopic dermatitis administered the bacteria Lactobacillus casei DN—114001. Polish J Microbiol. 2011;60(4):329–33. 22390068

[49] Shanahan L, Zucker N, Copeland WE, Costello EJ, Angold A. Are children and adolescents with food allergies at increased risk for psychopathology? J Psychosom Res. 2014;77(6):468–73. doi: 10.1016/j.jpsychores.2014.10.005 25454290 10.1016/j.jpsychores.2014.10.005 PMC4307934

[50] Patten SB, Williams JVA. Self-Reported Allergies and Their Relationship to Several Axis I Disorders in a Community Sample. Int J Psychiatry Med. 2007; 1;37(1):11–22. doi: 10.2190/L811-0738-10NG-7157 17645194 10.2190/L811-0738-10NG-7157

[51] Mascola AJ, Bryson SW, Agras WS. Picky eating during childhood: A longitudinal study to age 11 years. 2011;11(4):253–7. 10.1016/j.eatbeh.2010.05.006 PMC2943861 20850060

[52] Bryant-Waugh R, Markham L, Kreipe RE, Walsh BT. Feeding and eating disorders in childhood. Int J Eat Disord. 2010;43(2):98–111. doi: 10.1002/eat.20795 20063374 10.1002/eat.20795

[53] Yoshizawa M, Tashiro M, Fukudo S, Yanai K, Utsumi A, Kano M, et al Increased Brain Histamine H1 Receptor Binding in Patients with Anorexia Nervosa. Biol Psychiatry. 2009;65(4):329–35. doi: 10.1016/j.biopsych.2008.08.012 18814859 10.1016/j.biopsych.2008.08.012

[54] Bollinger ME, Dahlquist LM., Mudd K., Sonntag C, Dillinger L, McKenna K. The impact of food allergy on the daily activities of children and their families. Annals of Allergy, Asthma & Immunology. 2017; 96(3), 415–21. 10.1016/S1081-1206(10)60908-8 16597075

[55] Primeau MN, Kagan R, Joseph L, Lim H, Dufresne C, Duffy C, et al The psychological burden of peanut allergy as perceived by adults with peanut allergy and the parents of peanut-allergic children. Clin Exp Allergy. 2000;30(8):1135–43. 10931121 10.1046/j.1365-2222.2000.00889.x

[56] Berstad A, Arslan G, Lind R, Florvaag E. Food hypersensitivity—Immunologic (peripheral) or cognitive (central) sensitisation? Psychoneuroendocrinology. 2005;30(10):983–9. doi: 10.1016/j.psyneuen.2005.04.010 15979811 10.1016/j.psyneuen.2005.04.010

[57] Bell IR, Schwartz GE, Peterson JM, Amend D. Symptom and personality profiles of young adults from a college student population with self-reported illness from foods and chemicals. J Am Coll Nutr. 1993; 1;12(6):693–702. 8294725 10.1080/07315724.1993.10718361

[58] Nowak-Wegrzyn A, Assa’ad AH, Bahna SL, Bock SA, Sicherer SH, Teuber SS. Work Group report: Oral food challenge testing. J Allergy Clin Immunol. 2009;123(6 SUPPL). https://doi.org/10.1016/j.jaci.2009.03.042. doi: 10.1016/j.jaci.2009.03.042 19500710 10.1016/j.jaci.2009.03.042

[59] Ogrodowczyk A, Wroblewska B, Markiewicz LH, Zakrzewska M. Possible immunological consequences of filaggrin gene mutation. A case study of a 3-year-old allergic girl. Cent J Immunol. 2013;38(3):403–7.

