# Supplementary material for: Increased prevalence of eating disorders as a biopsychosocial implication of food allergy
Source: PLoS One. 2018 Jun 26;13(6):e0198607. doi: 10.1371/journal.pone.0198607 (PMC6019672; doi:10.1371/journal.pone.0198607)
Supplement: S1 Table — (DOCX) [file pone.0198607.s001.docx]

**S1 Table. Inclusion-exclusion criteria and specified assessments**

| **Inclusion criteria** | **Exclusion criteria** | **Specified assessments** |
| --- | --- | --- |
| Inclusion criteria for the allergic group in this study were:  **1)** manifestations specific for food allergic disease    **2)** confirmed status by immunological tests, with increased levels of E class antibodies and inflammatory cytokines  **3)** defined family history, where positive family history was defined as at least one biological parent having allergic symptoms, and a negative family history was defined as both biological parents with no developed allergy. | Individuals were excluded from this study if:  **1)** they were diagnosed as asthmatics, atopic (AD) or allergic rhinitis sufferers (AR) but not FA sufferers  **2)** they had positive history of cancer, or inflammatory bowel disease with acute or a chronic diarrhoea at the time of recruitment or prospectively during the 5-year follow-up    **3)** they confirmed neurological, psychiatric or systemic illness that might impact cognition or might interfere with longitudinal follow-up at the time of recruitment or prospectively during the 5-year period. | **1)** The normal body mass was stated for the weight for age (W/A) and weight for height (W/H) z-score 0 to ±2, whereby -2 to -3 indicated moderate underweight and <-3 z-scores indicated severe underweight. Conversely, overweight was defined as having W/A and W/H of >+2 z-scores and obesity as >+3 z-scores. Also the height for age (H/A) *z*-score was taken into consideration. |
