# Supplementary material for: Increased prevalence of eating disorders as a biopsychosocial implication of food allergy
Source: PLoS One. 2018 Jun 26;13(6):e0198607. doi: 10.1371/journal.pone.0198607 (PMC6019672; doi:10.1371/journal.pone.0198607)
Supplement: S2 Table — (DOCX) [file pone.0198607.s002.docx]

**S2 Table. Comprehensive characteristics of the studied underage population**

| **No.** | **Features** | **The description** |
| --- | --- | --- |
| **a.** | **The occurrence of menstruation** | Out of the 100 initially included female participants, 73 were premenarchal, and 27 reported a regular menstrual cycle. Out of the 79 female participants included in the follow-up phase of the study, 57 were premenarchal, 19 reported a regular menstrual cycle, and 3 were allergic participants who were classified as lean and reported a loss of menses for the last two months. |
| **b.** | **Changes in the population structure during the research** | Five participants from the allergic group were excluded from the experiment because they met the exclusion criteria (inflammatory bowel disease), seven participants developed tolerance, while three participants resigned from the next stage of the study despite persisting symptoms. In the control group, eight individuals withdrew from the study, and one participant was excluded because of a developed allergy to peanuts. |
| **c.** | **Birth method** | regardless of the group, natural birth was dominant; although in the allergic group (FA+), this method was declared 5% more often than in the control. |
| **d.** | **New-born feeding type** | 51.4% of allergic children were at least temporarily breastfed (for the first 6 months of life) compared to the control group where this breastfeeding was declared by 82.2% of participants. In the formula-fed group, there was a significantly higher proportion of individuals indicating a predisposition for eating abnormalities, both in control (p=0.0078) and allergic groups (p=0.0012). |
| **e.** | **The place of residence** | In the recruitment phase of the study, over 82% of the participants lived in the city; however, in the 5-year follow-up, a significant (*p*=0.013) increase in the number of participants who migrated to rural areas was observed |
| **f.** | **Profiles of food specific antibodies in sera of different groups of patients** | The most frequently determined polyallergy in the analysed FA+ group was combination of soybean (fl4), milk (f2), wheat (f4) and potato (f35). The most severe reactions were caused by wheat (f4), milk (f2), peanut (fl 3), codfish (f3) and celery (f85). In the group of patients suffering from FA+M, the most common allergies were to combinations of egg white (fl), milk (f2), peanut (fl3), wheat (f4), potatoes (f35) and birch (t3) |
