# Supplementary material for: Increased prevalence of eating disorders as a biopsychosocial implication of food allergy
Source: PLoS One. 2018 Jun 26;13(6):e0198607. doi: 10.1371/journal.pone.0198607 (PMC6019672; doi:10.1371/journal.pone.0198607)
Supplement: S1 Text — (DOCX) [file pone.0198607.s003.docx]

**S1 Text. Characteristic of the ED prevalence tests**

Both tests are based on questions with dichotomous response options (yes/no). In the SCOFF test, each affirmative answer was scored as 1 point. If two or more of the questions were affirmed, the SCOFF test assessment was considered positive and was defined as indicative of an ED. In the current sample, the SCOFF test revealed an internal consistency of α=0.76 and a sensitivity of 89%. In the later period, results were verified using The Eating Attitudes Test (EAT-8) as a second, recommended reference tool for detecting ED. EAT-8 revealed a high applicability for different ages and sexes as well as good psychometric properties. EAT-8 revealed an internal consistency of α=0.83 and sensitivity of 94%. Two cut-off points for participants (male: 2 points and female: 3 points) in EAT-8 were applied.
