# Supplementary figures and images for: Increased prevalence of eating disorders as a biopsychosocial implication of food allergy
Source: PLoS One. 2018 Jun 26;13(6):e0198607. doi: 10.1371/journal.pone.0198607 (PMC6019672; doi:10.1371/journal.pone.0198607)

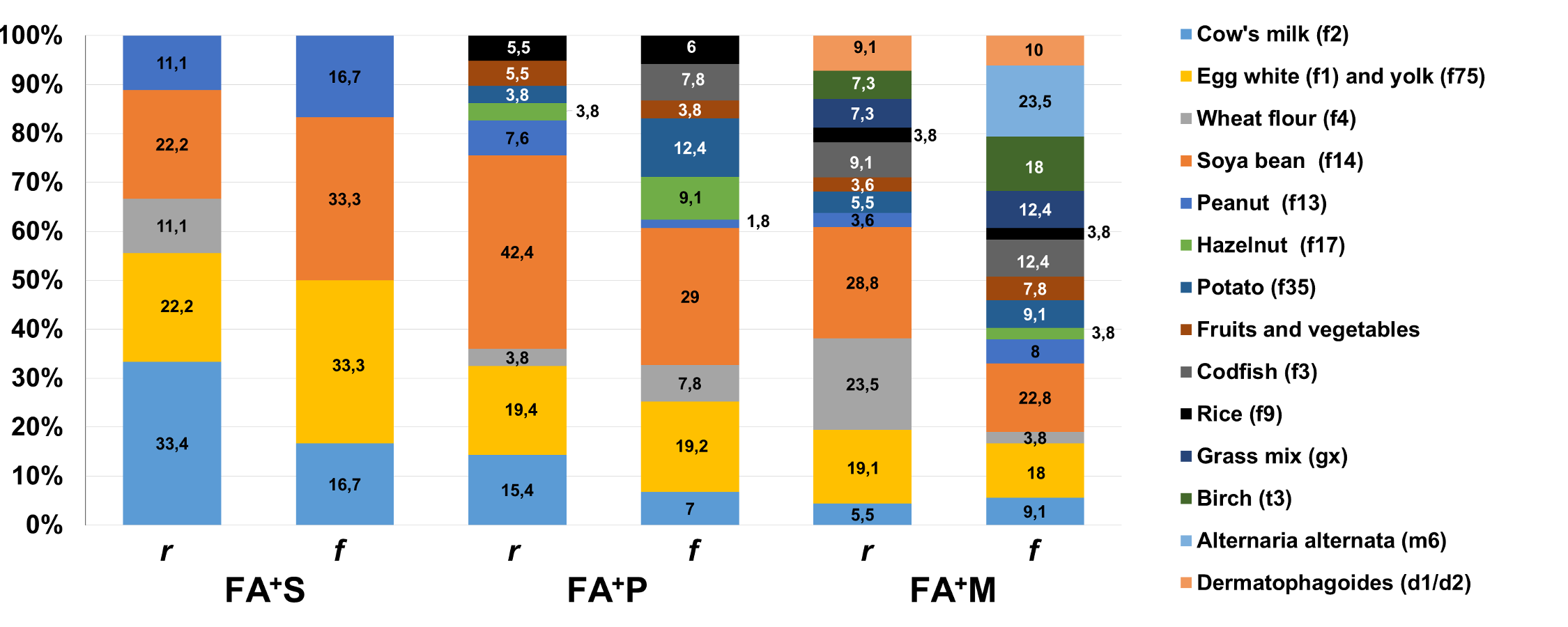

Supplement: S1 Fig — (TIF) [file pone.0198607.s004.tif]
